# Oral, Nasal and Pharyngeal Exposure to Lipopolysaccharide Causes a Fetal Inflammatory Response in Sheep

**DOI:** 10.1371/journal.pone.0119281

**Published:** 2015-03-20

**Authors:** Gunlawadee Maneenil, Matthew W. Kemp, Paranthaman Senthamarai Kannan, Boris W. Kramer, Masatoshi Saito, John P. Newnham, Alan H. Jobe, Suhas G. Kallapur

**Affiliations:** 1 Division of Pulmonary Biology, Cincinnati Children’s Hospital Medical Center, University of Cincinnati School of Medicine, Cincinnati, Ohio, United States of America; 2 School of Women’s and Infants’ Health, The University of Western Australia, Perth, Australia; 3 Department of Pediatrics, School of Oncology and Development Biology, Maastricht University Medical Center, Maastricht, Netherlands; 4 Department of Perinatal Medicine, Tohoku University Hospital, Sendai, Japan; 5 Department of Pediatrics, Faculty of Medicine, Prince of Songkla University, Thailand; University of Pittsburgh, UNITED STATES

## Abstract

**Background:**

A fetal inflammatory response (FIR) in sheep can be induced by intraamniotic or selective exposure of the fetal lung or gut to lipopolysaccharide (LPS). The oral, nasal, and pharyngeal cavities (ONP) contain lymphoid tissue and epithelium that are in contact with the amniotic fluid. The ability of the ONP epithelium and lymphoid tissue to initiate a FIR is unknown.

**Objective:**

To determine if FIR occurs after selective ONP exposure to LPS in fetal sheep.

**Methods:**

Using fetal recovery surgery, we isolated ONP from the fetal lung, GI tract, and amniotic fluid by tracheal and esophageal ligation and with an occlusive glove fitted over the snout. LPS (5 mg) or saline was infused with 24 h Alzet pumps secured in the oral cavity (n = 7–8/group). Animals were delivered 1 or 6 days after initiation of the LPS or saline infusions.

**Results:**

The ONP exposure to LPS had time-dependent systemic inflammatory effects with changes in WBC in cord blood, an increase in posterior mediastinal lymph node weight at 6 days, and pro-inflammatory mRNA responses in the fetal plasma, lung, and liver. Compared to controls, the expression of surfactant protein A mRNA increased 1 and 6 days after ONP exposure to LPS.

**Conclusion:**

ONP exposure to LPS alone can induce a mild FIR with time-dependent inflammatory responses in remote fetal tissues not directly exposed to LPS.

## Introduction

Chorioamnionitis is commonly associated with preterm delivery [1]. Chorioamnionitis can increase the expression of cytokines and chemokines within the gestational tissues and stimulate the production of prostaglandins, causing uterine contraction and preterm delivery [2]. The fetus can respond to infection and inflammation in the amniotic fluid with a fetal inflammatory response (FIR) that can be detected by increased IL-6 levels in cord plasma or by inflammation in the umbilical cord, termed funisitis [3,4]. This FIR is associated with increased risks of adverse postnatal outcomes such as necrotizing enterocolitis, postnatal sepsis, bronchopulmonary dysplasia, and brain injury [3,5–8]. In animal models of pregnancy, intra-amniotic injection of pro-inflammatory mediators or live organisms causes chorioamnionitis, preterm labor, and FIR with complex tolerance-type immune responses [9–13].

The fetal responses to inflammatory stimuli in amniotic fluid result from direct exposure of fetal epithelial surfaces—the chorioamnion, the fetal skin, the gut, and the lungs. The range of organ responses to LPS or *Ureaplasma* induced chorioamnionitis is best characterized in fetal sheep [10,14–17]. Both mediators cause skin inflammation, lung inflammation that progresses to induced lung maturation, and an arrest in gut maturation with local mild inflammation [10,14,18,19]. Selective exposures of the fetal lung, gut, or skin to agonists such as LPS or interleukin (IL)-1α cause distinct time dependent inflammatory changes in the liver, thymus, and white cell counts in the blood, indicating systemic inflammation [10]. In a previous study, selective exposures to the gut or lung were achieved by ligating the trachea or esophagus with organ targeted exposure to the agonists, while a selective skin/chorioamnion exposure was achieved by occluding the mouth and nose of the fetus [20]. For these previously reported experiments, the oral, nasal, and pharyngeal (ONP) epithelial and lymphoid tissues were excluded from agonist exposure. Thus the role of the ONP to initiate a FIR remains unexplored. The ONP tissues contribute a large surface area to the mucosal immune system, but their potential contribution to a FIR has not been assessed experimentally. The fetus swallows and breathes with the movement of large volumes of amniotic fluid over ONP surfaces [21]. Therefore, we hypothesized that exposure of the ONP only to LPS would cause a FIR that might have different characteristics from the other organ exposures.

## Materials and Methods

### Animals

All procedures with animals were at The University of Western Australia (Perth, WA) following review and approval by the animal care and use committees of The University of Western Australia and Cincinnati Children’s Hospital (Cincinnati, OH). Date bred Merino ewes with singleton pregnancies were randomized prior to surgery to receive either: i) 1 or 6 days saline; or ii) 1 or 6 days LPS. Ewes were pre-medicated with an intra-muscular (IM) injection of buprenorphine (0.02 mg/kg) and acepromazine (0.01 mg/kg) for at least 30 minutes before induction of anesthesia with an intravenous (IV) bolus of midazolam (0.25 mg/kg) and ketamine (5 mg/kg). Ewes were intubated and maintained on intermittent positive-pressure ventilation and anesthesia using inhaled isofluorane. Heart rate, venous and arterial pressure (mmHg), end-tidal CO_2_ and SpO_2_ were constantly monitored. Following sedation, preparation of the abdomen, and anesthesia, sterile fetal surgery was performed to isolate the ONP [20]. The proximal esophagus and trachea were isolated through a neck incision. The esophagus was ligated, and the trachea was cannulated with a large bore catheter connected to a 2 L silastic bag to permit fetal lung fluid to flow into the bag [20]. The proximal trachea was ligated. Each animal had a 2 mL 24 h osmotic pump (Alzet, Inc., Chicago, IL), secured with a suture in the oral cavity. A latex glove was then glued and sutured over the snout to separate the ONP from the amniotic fluid. The pump delivered 5 mg Escherichia Coli LPS serotype 055:B5 (Sigma-Aldrich, St. Louis, MO) or saline to the oral cavity over 24 h. Some of the control surgical animals were used in previous reports [20,22]. The dose of 5 mg was used in this study based on reported fraction of amniotic fluid volume swallowed by the fetus and our reports of 10 mg LPS given by intraamniotic injection (non surgical animals) needed to initiate a FIR [10,12,20,21].

The surgeries were performed 1 or 6 days before delivery. Ewes were delivered operatively at 123±2 days gestational age and euthanized with an intravenous injection of pentobarbital (100 mg/kg). Fetuses were also euthanized with an overdose of pentobarbital. Each fetus was weighed and fetal cord blood was collected for plasma, cell counts (VetPath, Perth, Western Australia), blood gas, and pH measurements. Following opening of the fetal chest, a deflation air pressure-volume curve was measured from a static inflation of 40 cmH_2_0 airway pressure [23]. Fetal tissues for protein or mRNA expression analysis were rapidly dissected and snap frozen in liquid nitrogen.

### Relative Quantification of mRNA Expression

Total RNA was isolated from fetal tissues homogenized in TRIzol (Life Technologies, Carlsbad, CA.) using a modified Chomezynski method, and mRNA quantitation was performed by real-time PCR [20]. The mRNA was reverse transcribed to yield a single-strand cDNA (Verso cDNA kit, Thermo Scientific, Waltham, MA.), which was used as a template with primer and Taqman probes (Applied Biosystems, Carlsbad CA.) specific to sheep sequences [11]. Gene expression was measured for surfactant proteins and cytokines: SP-A, SP-B, SP-D, IL-1β, IL-6, IL-8, TNF-α, monocyte chemotactant protein-1 (MCP-1), serum amyloid protein A3, and C-reactive protein. The values for each cytokine were normalized to internal 18S rRNA values. Final expression data are represented as fold increase over the control values.

### Fetal plasma cytokines and protein

Concentrations of fetal plasma cytokines were measured as previously described using sandwich enzyme-linked immunosorbent assays (ELISA) employing the following antibodies: IL-1β (coating antibody, rabbit anti-ovine IL-1β. Guinea pig anti-ovine IL-1β primary antibody [Both Seven Hills Bioreagents, Cincinnati, OH.]); IL-6 (coating antibody, mouse anti-ovine IL-6 [MAB 1004, Millipore, Billerica, MA.] Rabbit anti-ovine IL-6 primary antibody [AB1839, Millipore, Billerica, MA.] The detection antibody for all the assays was an appropriate specific HRP-conjugated antibody. The detection limits and the dynamic range of measurement were: IL-1β—0.2–12.0 ng/ mL, IL-6—0.2–12.0 ng/mL. The correlation coefficient was 0.94–0.99 for all assays. Plasma haptoglobin levels were measured ELISA following the manufacturer’s instruction (catalog no. E-35HPT; ICL, Portland, OR).

### Statistical Analyses

All values are expressed as mean ± standard deviation. Comparisons between groups were made with two-tailed unpaired t-tests. Non-parametric data were tested for significance with Mann-Whitney tests. Statistical analysis was performed by GraphPad Prism v5.0. Significance was accepted at p<0.05.

## Results

### Outcomes at Delivery and Cells in Cord Blood

None of the fetuses died prior to delivery and there were no differences in cord blood pH and gas values between groups. The fetuses exposed to LPS had viscous secretions in the ONP. Fetal weights and white blood cell counts in umbilical venous blood are given in Table 1. One day after ONP exposure to LPS, the fetal circulating WBC decreased significantly compared with the control group. In contrast, the total count increased at 6 days after LPS exposure, relative to saline control. Neutrophil lymphocyte and monocyte counts also significantly increased in the 6 days LPS group. Thus, ONP exposure to LPS caused a systemic FIR as indicated by changes in peripheral WBCs.

**Table 1:** Physiological variables at delivery and cells in cord blood.

	Control 1 d and 6 d(Saline ONP)	1 d LPS in ONP	6 d LPS in ONP
n	8	7	8
GA, days	124±0.8	121±3.4	124±0.5
Birth weight, kg	2.9±0.3	3.0±0.2	2.8±0.3
Total WBC, 10^9^/L	2.7±0.7	1.7±0.7*	6.8±3.1*
Neutrophils, 10^9^/L	0.9±0.6	0.7±0.5	3.4±1.7*
Lymphocytes, 10^9^/L	1.7±0.5	1.3±0.5	2.8±0.9*
Monocytes, 10^9^/L	0.1±0.1	0.1±0.5	0.7±0.5*
Lung weight to BW ratio (g/kg)	34.0± 7.8	34.2±4.7	37.2±11.5

Values are mean ± SD. BW, body weight; WBC, white blood cells; GA, gestational age

*p<0.05 vs. Control Saline

### Response in Fetal Lungs

**Fig 1:**
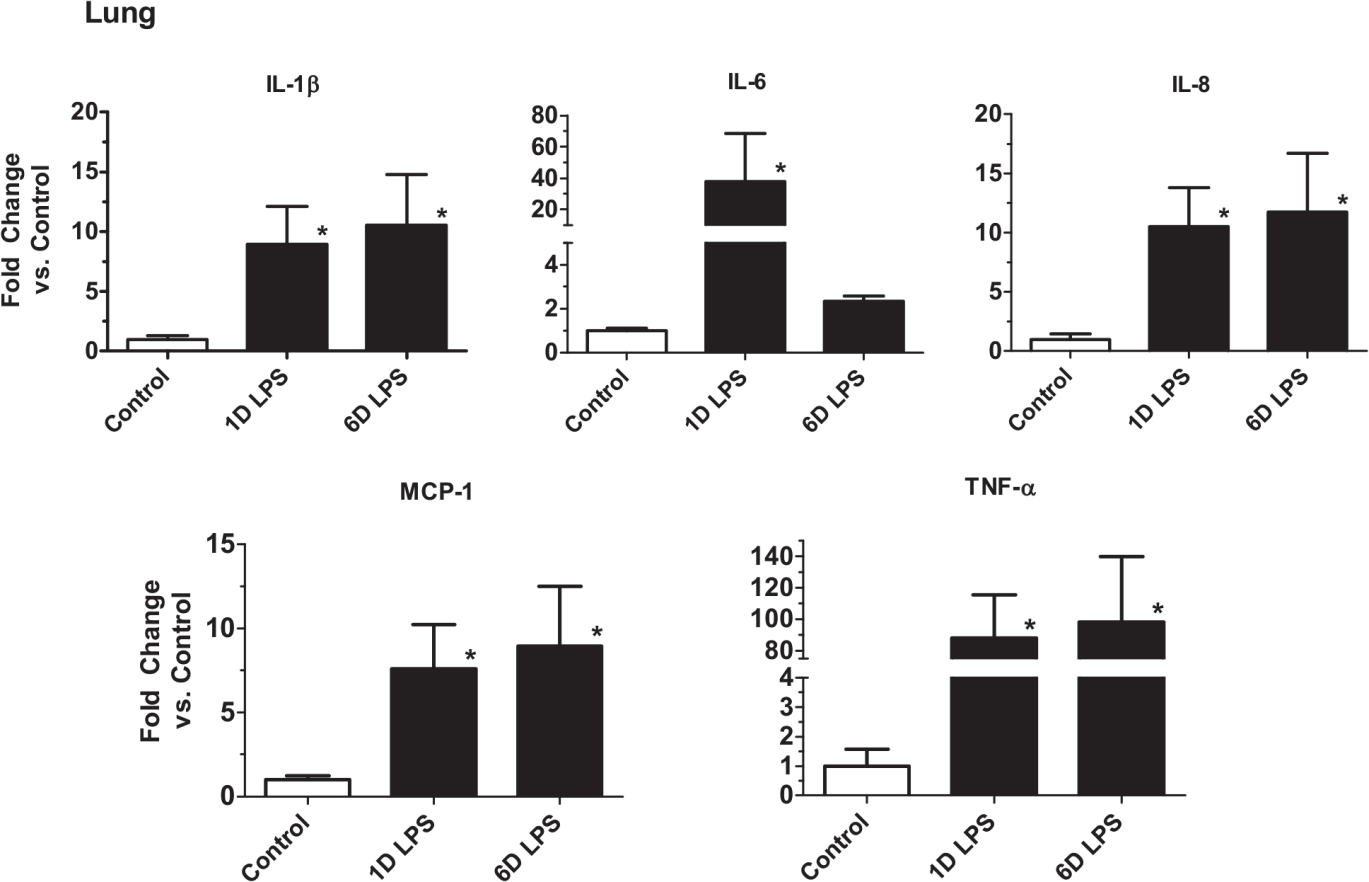
ONP exposure to LPS induced lung cytokine mRNA expression. Quantification of messenger RNAs for IL-1β, IL-6, IL-8, MCP-1 and TNF-α was performed by PCR using sheep-specific primers. Each cytokine was normalized to 18S ribosomal protein mRNA (internal control), and levels for each group were expressed as fold increase relative to controls. Cytokine expression was increased in 1 and 6 days LPS group. (*p<0.05 vs. Control Saline)

**Fig 2:**
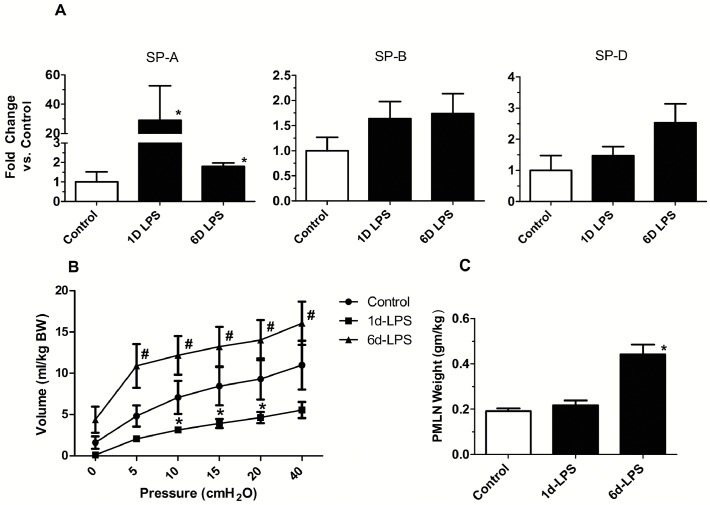
ONP exposure to LPS increased indicators of lung maturation. **(A)** Quantification of messenger RNAs for surfactant protein A (SP-A), surfactant protein B (SP-B) and surfactant protein D (SP-D) was performed by PCR using sheep-specific primers. SP-A mRNA levels were significantly increased in 1 and 6 days LPS group. SP-B and SP-D mRNA levels were increased but not significantly. **(B)** Pressure-Volume curve of the sheep after 1 day of LPS exposure was decreased compared with control group. Lung volume increased at 6 days relative to the 1 day LPS exposure. **(C)** ONP exposure to LPS increased the weight of the posterior mediastinal lymph node at 6 days. (*p<0.05 vs. Control Saline, ^#^ *p*<0.05 vs 1 day LPS)

**Fig 3:**
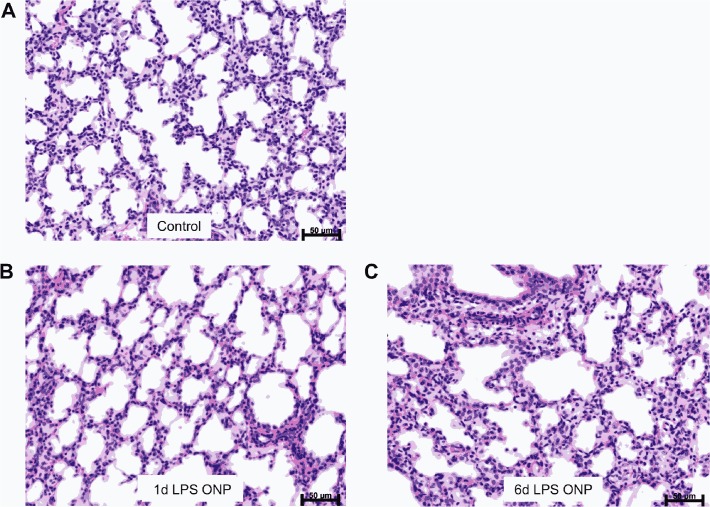
Fetal lung histology following LPS exposure. Representative photograph shows mild interstitial thickening in 1 day and 6 days LPS groups **(B, C)** compared to the saline control group **(A).** Scale bar represents 50 μm. Magnification x20

Pulmonary inflammation resulting from ONP exposure to LPS was assessed by measuring mRNA expression for pro-inflammatory cytokines. mRNA for IL-1β, IL-6, IL-8, MCP-1 and TNF-α in the fetal lung increased 1 day and 6 days after exposure to LPS relative to saline control (Fig. 1). SP-A mRNA in the lung increased significantly 1 day and 6 days after LPS exposure. SP-B and SP-D mRNA levels increased, but not significantly (Fig. 2A). Lung gas volume measured at 10, 15 and 20 cmH_2_O decreased significantly 1 day after LPS exposure relative to saline control (Fig. 2B). In the 6 day LPS group, lung gas volumes increased relative to the 1 day LPS group and tended to increase relative to saline control. The lung weight to body weight ratio for the 6 days LPS group increased but not significantly compared to saline control (Table 1). To determine the effect of LPS on lung structure, we evaluated histology with hematoxylin and eosin staining. Compared to controls, mild interstitial thickening was noted in the 1 day and 6 days LPS group (Fig. 3).

### Inflammatory markers in the blood

**Fig 4:**
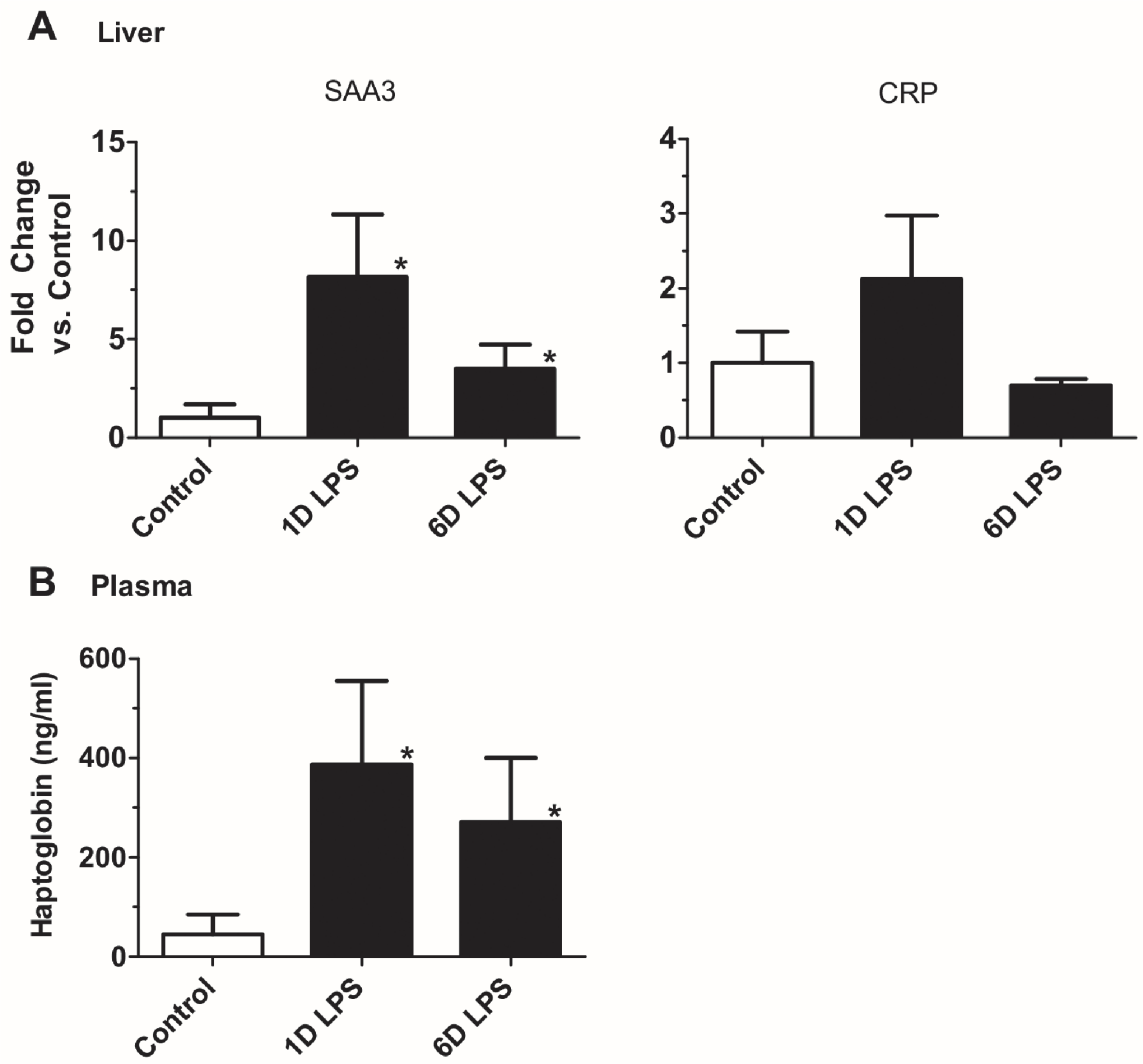
Acute phase protein in liver and plasma. **(A)** Quantification of messenger RNAs for SAA3 and CRP was performed by PCR using sheep-specific primers. Each mRNA was normalized to 18S ribosomal protein mRNA (internal control), and levels were expressed as fold increase relative to controls. SAA3 was significantly increased in 1 and 6 days LPS group. **(B)** Plasma haptoglobin levels were measured by ELISA. Haptoglobin levels were significantly increased at 1 day and 6 days after LPS exposure. (*p<0.05 vs. Control Saline)

The acute phase plasma protein haptoglobin is a marker for systemic inflammation. Relative to controls, plasma haptoglobin levels increased significantly 1 day and 6 days after LPS exposure (Fig. 4B). In contrast plasma cytokines, IL-1β and IL-6 concentration did not increase in the fetal plasma of control and experimental groups (data not shown).

### Posterior mediastinal lymph node (PMLN)

We evaluated the weight of PMLN because this node receives lymphatic flow from the lung and gastrointestinal tract [24]. The PMLN to body weight ratio for the 6 days LPS group increased significantly relative to the control group (Fig. 2C).

### Inflammation in the Fetal Liver

Hepatic inflammation was assessed by measuring mRNA expression for the acute phase response proteins, serum amyloid protein A3 (SAA3) and C-reactive protein (CRP). SAA3 mRNA expression significantly increased for 1 day and 6 days LPS compared to saline control. CRP trended to increase for 1 day LPS group (Fig. 4A).

## Discussion

A mechanistic question is how inflammatory products in the amniotic fluid (AF) cause inflammation and injury in distant fetal organs such as the brain. This is a complex question since the microorganisms in chorioamnionitis are largely restricted to the AF and not systemically disseminated because <5% of preterm infants with histologic chorioamnionitis have early onset sepsis [25,26]. In previous studies with fetal sheep, an intravascular injection of 6 μg LPS caused profound hypotension and acidosis in preterm sheep, but an intraamniotic dose of up to 100 mg (10,000 fold excess of IV dose) was well tolerated without hypotension in the fetus [27,28]. These data strongly argue for a strict compartmentalization of inflammatory mediators to the AF with secondary signaling to the fetus via organs in direct contact with the amniotic fluid. In a series of experiments, we have asked how each of the organs in contact with the amniotic fluid signals fetal inflammatory responses [10,14,22]. The fetus actively swallows amniotic fluid and therefore products of inflammation within the AF contact the rich lymphoid tissue within the oropharyngeal nasal (ONP) cavity. We report the novel observation that selective ONP exposure to LPS causes systemic effects with changes in WBC, neutrophils, lymphocytes and monocytes in cord blood, increases in plasma haptoglobin level, increases in posterior mediastinal lymph node (PMLN) weight at 6 days and increases mRNA expression of SAA3 in liver. Furthermore, the effects include pulmonary inflammation, changes in the pressure volume curve, interstitial thickening of lung tissue and increases in SP-A mRNA.

The characteristic of the FIR caused by ONP exposure needs to be interpreted within the context of other sheep models of chorioamnionitis and FIR. Intraamniotic injection of pro-inflammatory mediators such as LPS, IL-1, *Ureaplasma parvum* or *Candida albicans* cause different “FIR phenotypes” [11,15,29]. LPS causes acute chorioamnionitis, striking lung inflammation and maturation and systemic inflammation in multiple organs including the skin, gut, liver and the immune system (spleen, thymus, lymph node) but no fetal death. Intraamniotic IL-1 causes lung inflammation and maturation comparable to LPS but without tolerance type immune response that occur with LPS [11,30]. In contrast *Ureaplasma parvum* infects the amniotic fluid, fetal membranes, and lung but with less systemic inflammation and inconsistent lung maturation [17]. The other extreme is *Candida albicans* which quickly colonizes the fetal skin, causes a consolidation pneumonia and if left untreated kills the fetus [29].

To better understand how the pro-inflammatory mediators cause organ responses in the fetus, we have used fetal surgery in a series of experiments planned prospectively to achieve targeted organ exposure [10,20]. Each of these experiments is self-contained in that the controls and experimental animals are contemporaneous. The controls for each of the organ exposures involve identical fetal surgeries as the experimental animals with the exception of infusion of LPS vs. saline. In initial experiments, exposure of the fetal lung to LPS replicated the lung inflammation and maturation response achieved by intraamniotic LPS, but isolation of the lung from the pro-inflammatory mediator prevented a lung response indicating that the lung effects are mediated by direct contact only [9]. Gut-only exposure to intraamniotic LPS or IL-1 caused inflammatory changes in the lung as well as a change in gut structure [22]. Lung or gut exposure also increased the size of posterior mediastinal lymph node and caused systemic immune effects [10]. More recently we learned that the fetal skin responds to intraamniotic LPS, *Ureaplasma parvum* or *Candida albicans* with local inflammation, but LPS exposure to the skin and chorioamnion only does not cause a large FIR [19,20]. The preterm fetal epithelium that includes the immature skin, the lung and the gut respond to exposure to pro-inflammatory mediators with fetal organ and immune system responses that differ in time after the exposure and the magnitude of the responses (see Table 2). Thus the current study adds to the developing information of a highly nuanced FIR after chorioamnionitis that has contributions from several fetal organs in contact with the amniotic fluid. The clinical implication is that amniotic cavity is likely a good therapeutic target for future anti-inflammatory treatment strategies as an adjunct to anti-microbials for chorioamnionitis.

**Table 2:** Summary of the characteristics of fetal inflammatory response (FIR): Contributions of different fetal organs.

	FIR in organs in contact with the amniotic fluid			FIR in organs not in contact with the amniotic fluid
Effect of LPS infusion in this tissue/organ	Lung	Gut	Skin/Amnion-Chorion	Blood/Liver/Spleen
Lung	+	+	?	++
Gut	−	+	?	±
ONP	+	?	?	+
Skin/Amnion-Chorion	−	−	+	+
Blood	fetal acidosis and death			

Based on the current study and references [10, 20, 22, 27].

Fetal exposure to LPS in the ONP induced a unique fetal inflammatory response. Lung cytokine mRNAs for IL-1ß, IL-8, MCP-1 and TNF-α had a persistent increase both at 1d and 6d after exposures, while IL-6 mRNA increased at 1d with a decrease at 6d. This pattern of persistent increase in cytokine mRNAs in the lung after ONP exposure is different from our previous results with lung only or intraamniotic LPS expousure (20). The precise reason for a brief early induction of IL-6 in the lung after ONP LPS exposure vs. persistent increase in other cytokines tested is not known. Similarly the increase in serum amyloid mRNA but not CRP mRNA in the liver after ONP exposure is also different from lung only LPS which induces both serum amyloid and CRP mRNAs in the liver. These results point to unique patterns of fetal inflammation based on the organ of LPS exposure.

This experiment to target exposure of the ONP epithelium to LPS is the last “organ targeting” in our series of experiments to define how the fetus senses the presence of proinflammatory mediators in amniotic fluid. The surface area of the ONP is large and rich in lymphoid tissue, and amniotic fluid passes across the ONP epithelium as the fetus breathes and swallows about half the amniotic fluid volume every 24 h [21]. The FIR response from ONP is distinct from LPS in amniotic fluid or a lung only LPS exposure. In general the magnitude of the lung inflammation was less compared to cytokine expression after intraamniotic LPS in non-surgical animals at 2 days [31], but the increased expression continued to 6 days which was not true for the intraamniotic LPS exposure or lung only exposure [20,31]. Also, the expression of TNF- α was greater for the ONP exposure than for intraamniotic or lung only exposure to LPS [9,20,31]. The lung maturation from ONP was less and inconsistent when compared to intraamniotic injection of LPS. We previously noted a decrease in lung volume 1 to 2 days after intraamniotic LPS [31], presumably from edema and inflammation, a response also detected with the ONP exposure. The increase in SP-A and the increase in the lung gas volume from 1 to 6 days indicate partial lung maturation, but the response of mRNA for the other surfactant proteins and the pressure-volume curves indicate an incomplete lung maturation response. We have not achieved large lung maturation responses without direct contact of the agonist with the fetal lung.

The increase in size of the PMLN did not occur until 6 days with the ONP exposure while amniotic fluid, gut or lung response increased PMLN weight within 1 to 2 days. Nevertheless, the ONP exposure did cause systemic responses in white blood cells, and a modest increase in plasma haptoglobin levels and SAA3 in the fetal liver. We conclude that the ONP epithelium is a component of the detection system for inflammatory mediators in amniotic fluid and is sufficient to initiate a mild FIR.
